# Current Trends in Mini-Clinical Evaluation Exercise in Medical Education: A Bibliometric Analysis

**DOI:** 10.7759/cureus.33121

**Published:** 2022-12-30

**Authors:** Ripudaman Sharma, Tarun Gupta, Tariq H Haidery, Siddhartha Sinha, Arvind Kumar

**Affiliations:** 1 Orthopaedics, GS Medical College and Hospital, New Delhi, IND; 2 Surgery, Hamdard Institute of Medical Sciences and Research, New Delhi, IND; 3 Medical Education, Hamdard Institute of Medical Sciences and Research, New Delhi, IND; 4 Orthopaedics, Hamdard Institute of Medical Sciences and Research, New Delhi, IND; 5 Orthopaedics, Maulana Azad Medical College, New Delhi, IND

**Keywords:** undergraduate, trends, training, specialty, postgraduate, medical education, mini-cex, clinical exercise, bibliometric

## Abstract

There has been emerging evidence supporting the mini-clinical evaluation exercise (mini-CEX) in various clinical specialties and settings. However, we need more clarity regarding the applicability of mini-CEX as an optimal assessment tool. Consequently, it has not been implemented on a wider scale, and several clinical specialties are yet to explore the benefits of mini-CEX. Therefore, we conducted a bibliometric analysis to investigate the publication trends of mini-CEX.

We searched the Web of Science database for mini-CEX-related original and review articles. The search results were analyzed for year-wise contribution, citation trends, contributing journals, contributing institutions, countries, authors, distribution of original/review articles, retrospective/prospective/laboratory/other types of studies, specialties covered, nature of medical education (undergraduate vs. specialty trainees), and clinical settings involved in the studies (single/multiple).

A total of 59 eligible articles (53 original and six review articles) were published between 1995 and 2022 in 35 different journals. The mean citations per year were 65.96 per year, and the mean citations per article per year were 2.34 citations per article per year. The articles published in *BMC Medical Education* and* Medical Teacher* were the highest in number. In total, 97 institutes contributed to the mini-CEX-related research, mostly from the University of Bern, Switzerland. There were 238 contributing authors, with Norcini JJ contributing the most number of articles. The remaining articles were 15 retrospective studies, one developmental study, six review articles, and three laboratory-based studies. The 50 non-laboratory studies involved students/trainees in medical and allied fields. Medicine was the most frequently covered specialty. The participants were mostly specialty trainees, followed by undergraduate medical students. Multiple settings were used in 38% of the reviewed studies and single in 16%.

The published articles have reduced impact and growth, as evidenced by low annual growth rates and citation trends. However, the available evidence was of reasonable quality considering the contribution from mostly prospective studies. Furthermore, it suggests considerable potential for further investigating the role of mini-CEX in clinical teaching.

## Introduction and background

In medical education, traditional clinical exercises (CEX) were designed to evaluate the performance of medical students and residents in real-life clinical scenarios, which was not feasible with conventional assessment methods such as written examinations and objective structured clinical examination (OSCE) [1]. The theme of CEX was based on bedside oral examination, in which an accessor evaluates the resident performing history and physical examination on the patient to reach a diagnosis. However, the major limitations of traditional CEX include prolonged time for assessment (approximately two hours) and the need for standardized evaluation [2,3]. Moreover, busy specialty clinics need an additional workforce, which is difficult to achieve and leads to quality compromise of the assessment [4]. Considering these shortcomings, the American Board of Internal Medicine introduced mini-CEX, a shorter version of CEX [5]. The mini-CEX has multiple advantages, which include a shorter assessment of 15-20 minutes, domain-focused scoring, objectiveness considering the nine-point scale for grading individual domains, and, most importantly, the possibility of multiple such assessments in the time required for single traditional CEX assessment [1,5,6]. Six domains are covered in mini-CEX, namely, interviewing skills, physical examination skills, professionalism, clinical judgment, organizing and efficiency, and counseling skills.

Clinical teaching and assessment quality has remained a constant concern in medical education [7]. Consequently, various teaching-assessment methods have been suggested to improve clinical learning among medical students and trainees [8]. The teaching methods include case-based learning, evidence-based medicine, problem-based learning, simulation-based learning, video-based learning, peer-assisted learning, observational learning, flipped classrooms, etc. While the assessment methods include written assessments, multiple choice questions, OSCE, short case assessments, long case assessments, log books, simulated patient surgeries, video-based assessments, simulators, self-assessments, peer assessments, and standardized patients.

There has been emerging evidence supporting the use of mini-CEX in various clinical specialties and settings [9]. However, considering its relatively new nature as a clinical assessment method and limited evidence in the literature, the outreach of mini-CEX as a standard assessment tool may be constrained. In addition, there are concerns regarding the validity of mini-CEX as an optimal assessment tool in different specialties and levels of expertise of the trainees [10,11]. A bibliometric analysis of the major articles can help understand the currently available evidence related to mini-CEX, especially the trends, type of research, specialty, settings, and popular themes, and help prepare future research strategies. We, therefore, conducted a bibliometric analysis to investigate the publication trends of mini-CEX.

## Review

**Figure 1:**
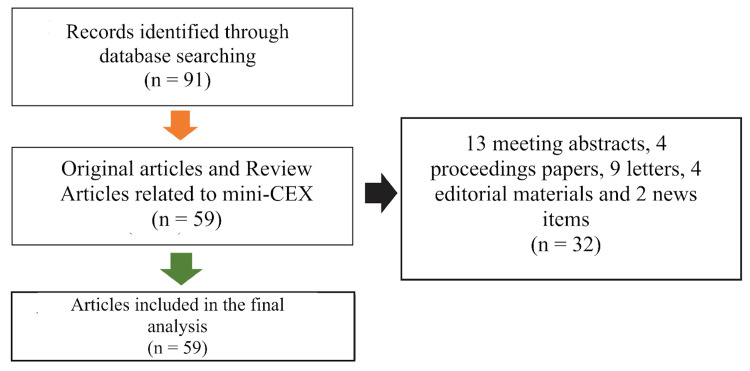
The search strategy of the current analysis of mini-CEX-related research. Mini-CEX = mini-clinical evaluation exercise

Two authors independently conducted a comprehensive search on the Web of Science database using the following keywords separated by the specified Boolean operators: mini CEX or mini-CEX or (mini clinical evaluation exercise) under the title field on August 10, 2022 (Figure 1). The results were cross-checked for discrepancies, and subsequent corrections were done if needed. We only included original articles and review articles in our analysis without any language or regional restrictions. The other article types, such as case reports, abstract-only publications, conference proceedings, letters, and editorials, were excluded. For quantitative analysis, the data were entered into the Bibliometrix R-package software (Source: https://www.bibliometrix.org) [12]. The eligible articles were analyzed for year-wise contribution, citation trends, contributing journals, contributing institutions and countries, and authors.

Additionally, we conducted a descriptive analysis using Microsoft® Excel, version 16.59. The continuous variables were expressed as mean, and the discrete variables were expressed as frequency distribution or proportions. We calculated the annual growth rate, i.e., the average number of articles contributed every year, overall citations, citations per article per year after publication, journals’ growth, i.e., the contribution of journals to the listed articles over the years, and authors productivity which included the overall contribution and productive years of the contributing authors. The quality of the selected articles was assessed through the distribution of original articles and review articles, retrospective/prospective/laboratory/other types of studies, specialties covered, nature of medical education (undergraduate vs. specialty trainees), and clinical settings involved in the studies (single/multiple).

Results

The search strategy resulted in 91 titles, of which 59 were included. The excluded articles included 13 meeting abstracts, four proceedings papers, nine letters, four editorials, and two news items. The articles were published between 1995 and 2022 and belonged to 35 journal sources.

Year-wise contribution

**Figure 2:**
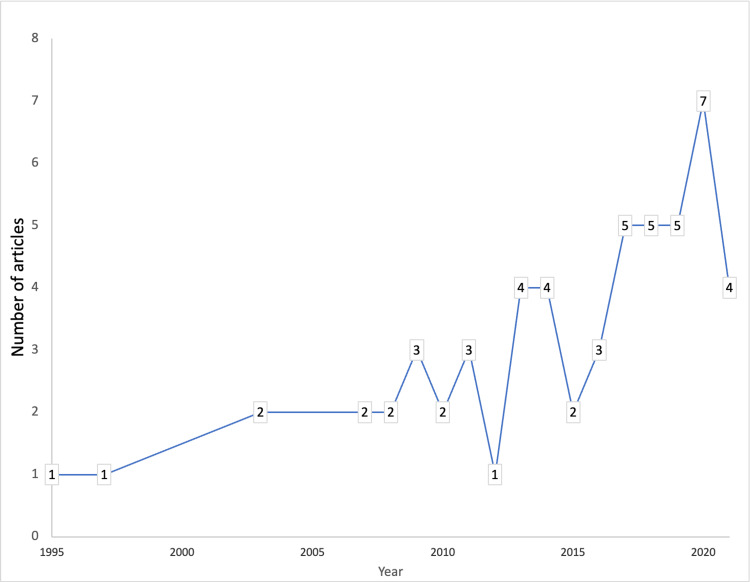
Year-wise contribution of the mini-CEX-related articles. Mini-CEX = mini-clinical evaluation exercise

The year-wise contributions to mini-CEX-related articles are shown in Figure 2. The annual growth rate was 4.15 articles per year. An increasing contribution of articles in recent years was observed, with 2020 being the most productive year.

Citation trends

**Figure 3:**
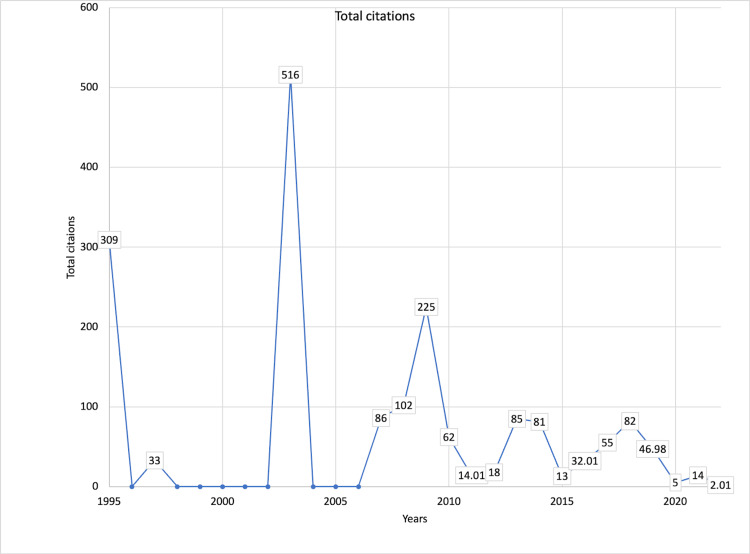
Year-wise overall citation count from mini-CEX-related articles. Mini-CEX = mini-clinical evaluation exercise

**Figure 4:**
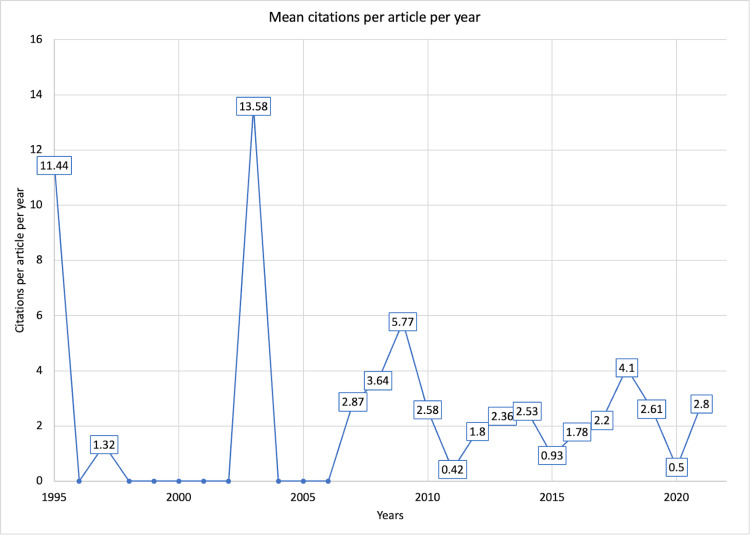
The mean citations per year of mini-CEX-related articles from the date of publication. Mini-CEX = mini-clinical evaluation exercise

The listed articles received a total of 1,781 citations. The articles published in 2003 received the highest number of citations (Figure 3). A similar trend was observed for citations contributed per article per year. The mean citations per year were 65.96 per year, and the mean citations per article per year were 2.34 citations per article per year (Figure 4). The top 10 cited articles are listed in Table 1.

**Table 1:** Top 10 cited articles related to mini-CEX. Mini-CEX = mini-clinical evaluation exercise

Article title	Authors	Citation count
The mini-CEX: a method for assessing clinical skills	Norcini et al. [1]	411
The mini-CEX (clinical evaluation exercise): a preliminary investigation	Norcini et al. [13]	309
Effect of rater training on reliability and accuracy of mini-CEX scores: a randomized, controlled trial	Cook et al. [14]	117
Construct validity of the miniclinical evaluation exercise (miniCEX)	Holmboe et al. [15]	105
Identifying the factors that determine feedback given to undergraduate medical students following formative mini-CEX assessments	Fernando et al. [16]	57
Does scale length matter? A comparison of nine- versus five-point rating scales for the mini-CEX	Cook et al. [17]	56
Implementing the undergraduate mini-CEX: a tailored approach at Southampton University	Hill et al. [18]	52
Validity, reliability, feasibility and satisfaction of the Mini-Clinical Evaluation Exercise (Mini-CEX) for cardiology residency training	Alves de Lima et al. [19]	48
The mini clinical evaluation exercise (mini-CEX) for assessing clinical performance of international medical graduates	Nair et al. [20]	45
Internal structure of mini-CEX scores for internal medicine residents: factor analysis and generalizability	Cook et al. [21]	45

Contributing journals

**Figure 5:**
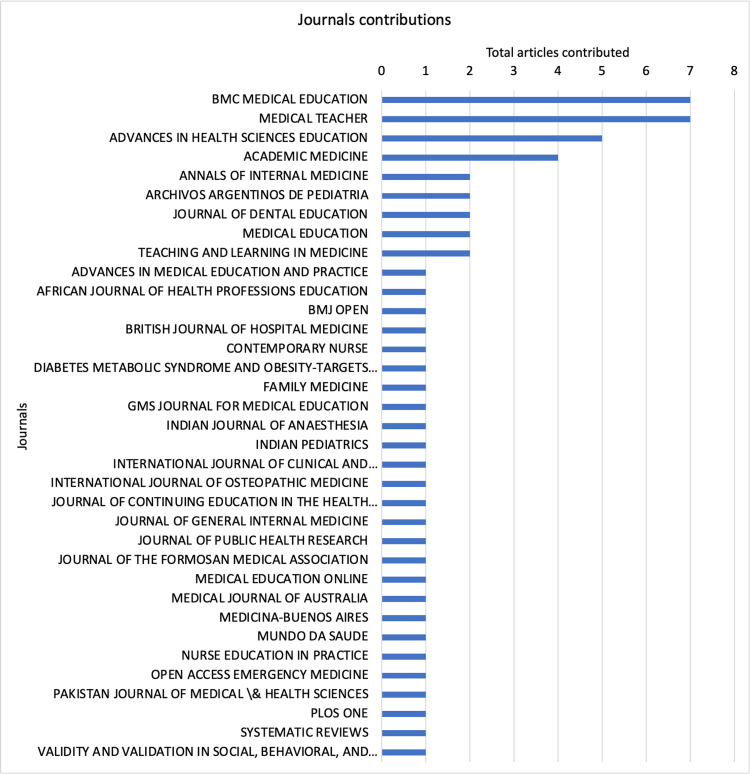
The contribution of different journals toward mini-CEX-related articles. Mini-CEX = mini-clinical evaluation exercise

**Figure 6:**
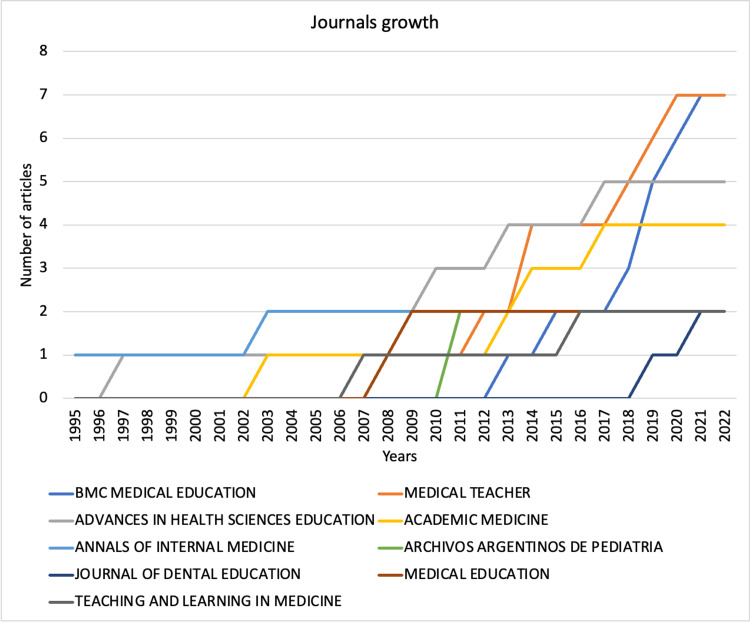
The cumulative contribution of mini-CEX-related articles from different journals. Mini-CEX = mini-clinical evaluation exercise

The 35 contributing journals and their article numbers are shown in Figure 5. The articles published by *BMC Medical Education* and *Medical Teacher* were the highest in number. The contribution growth curve also suggests cumulative growth in these journal contributions over the years (Figure 6).

Contributing institutions and countries

**Figure 7:**
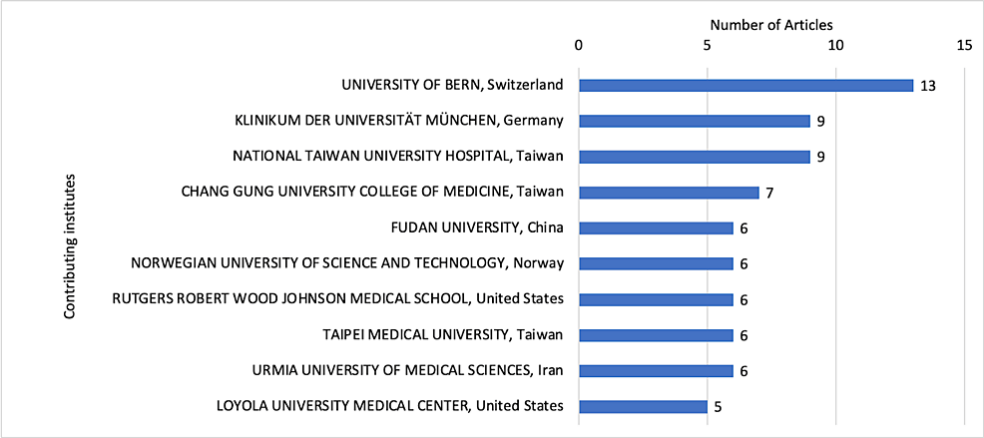
Top universities contributing to mini-CEX-related articles. Mini-CEX = mini-clinical evaluation exercise

**Figure 8:**
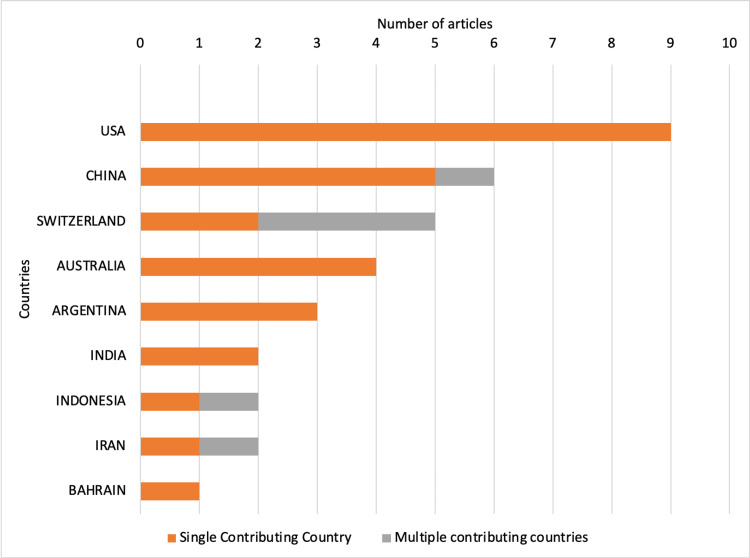
Top countries contributing to mini-CEX-related research. Mini-CEX = mini-clinical evaluation exercise

In total, 97 institutes contributed to the mini-CEX-related research. The maximum institutional occurrences were of the University of Bern, Switzerland (Figure 7). However, the corresponding authors from the United States contributed the most number of articles (Figure 8). Most international collaborations were observed in publications from Switzerland.

Authors’ contribution

**Figure 9:**
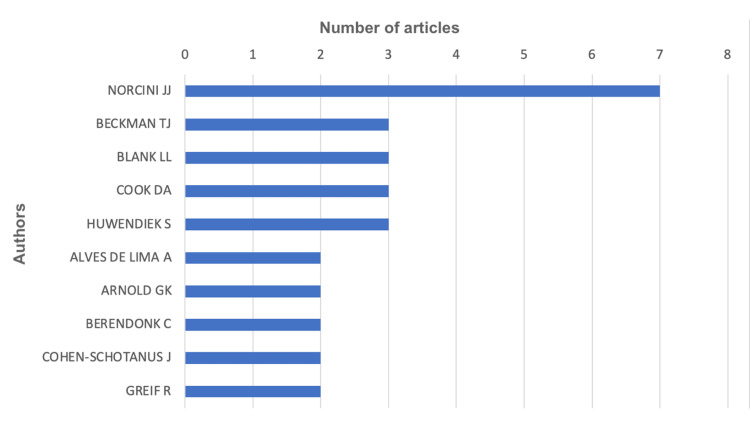
The top contributing authors toward mini-CEX-related research. Mini-CEX = mini-clinical evaluation exercise

**Figure 10:**
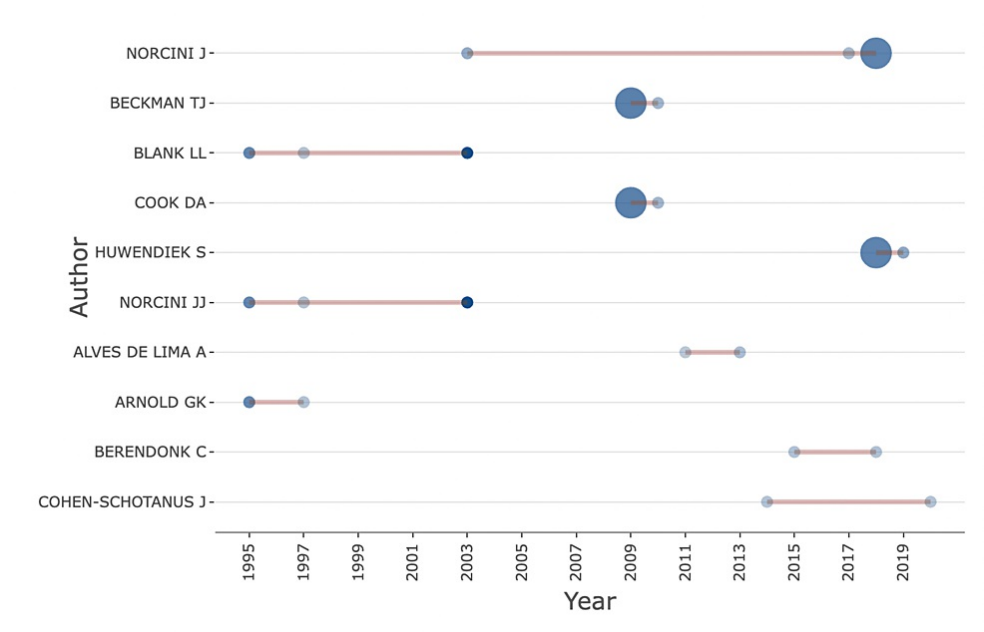
Authors’ productivity towards mini-CEX-related research. Mini-CEX = mini-clinical evaluation exercise

There were 238 contributing authors. The authors with at least two contributed publications are listed in Figure 9. Norcini JJ had the highest number of published papers on mini-CEX and the longest duration of productive years (Figure 10). His last publication was in the year 2018.

Quality of evidence

**Figure 11:**
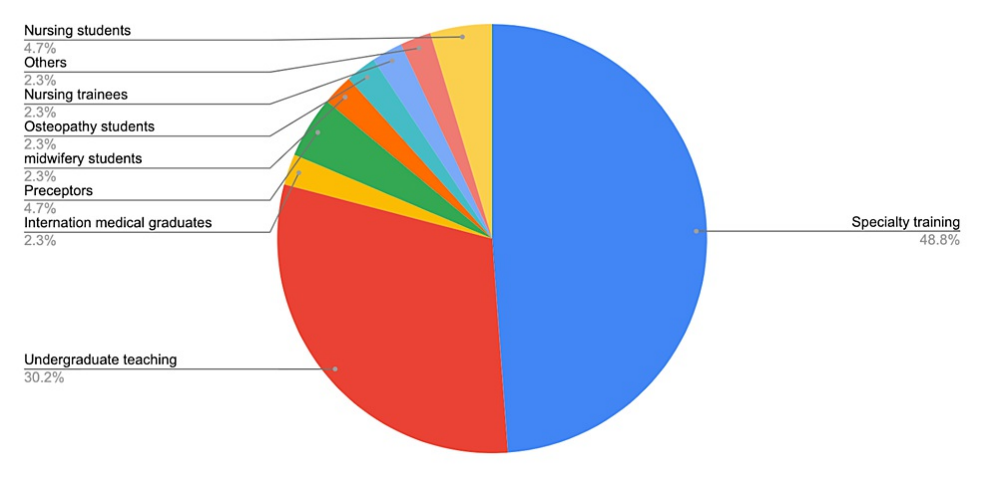
The distribution of different types of participants in the mini-CEX articles. Mini-CEX = mini-clinical evaluation exercise

**Figure 12:**
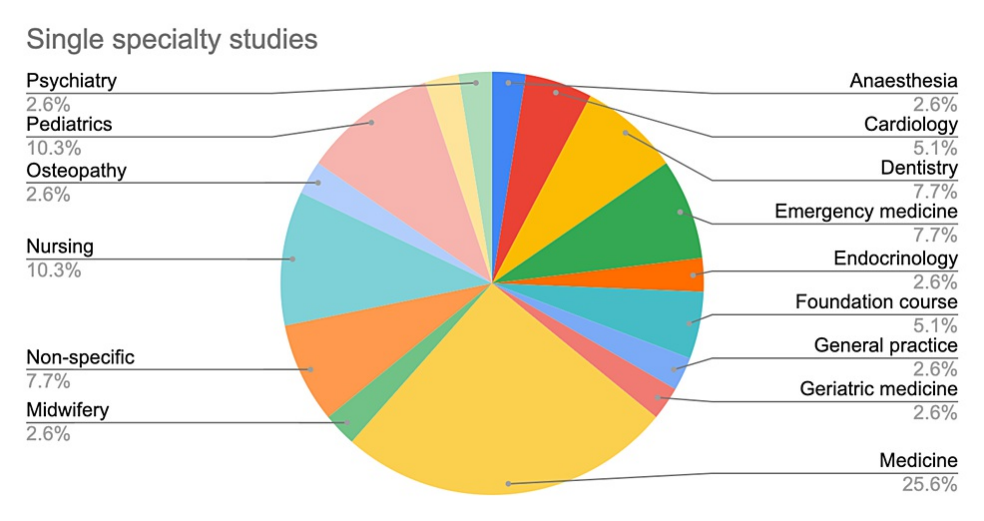
The distribution of specialties covered in studies involving a single specialty.

**Figure 13:**
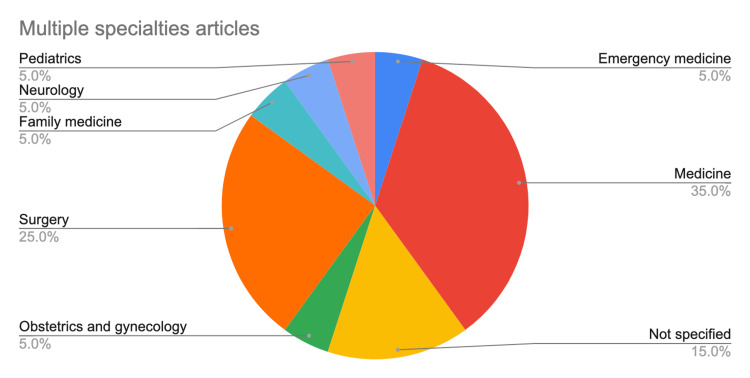
The distribution of specialties covered in studies involving multiple specialties.

There were 34 prospective studies, including four randomized controlled trials. The remaining articles were 15 retrospective studies, one developmental study, six review articles, and three laboratory-based studies. All review articles were systematic reviews/meta-analyses. Among the 53 non-review articles, 39 covered single specialties, while 14 covered multiple specialties. Fifty non-laboratory studies involved students/trainees of medical and allied fields, the details of which are provided in Figure 11. The mini CEX was implemented in a wide range of specialties, with medicine being the most frequently covered specialty (Figures 12, 13). The participants were mostly specialty trainees, followed by undergraduate medical students. Multiple settings were used in 38% of the reviewed studies, and single settings in 16%. The settings were not specified in the remaining studies.

Discussion

The findings of the current bibliometric analysis suggest that the evidence related to mini-CEX is still limited despite its implementation over two decades ago. The annual contribution of mini-CEX-related research is also low, with just more than four articles contributed every year on average. Fortunately, the number has been higher in recent years. The research productivity concerning mini-CEX also needs to improve, with just 66 citations contributed each year and fewer than three citations per article per year. The *BMC Medical Education* and *Medical Teacher* are well-recognized journals publishing medical education articles, and the same was reflected in our findings, with most articles belonging to these sources. The institutes contributing to the mini-CEX articles were mostly from developed/advanced/economically progressing countries, suggesting the availability of resources and infrastructure needed for mini-CEX implementation and overall expenditure in medical education. However, international collaborations among the institutes were low, with none for articles originating from the United States. Norcini JJ has published most articles on mini-CEX with the most extended active period and has also been productive in recent years. The quality of evidence, although limited, is superior, considering that more than half of the listed articles belonged to prospective studies and four randomized controlled trials. The review articles were all systematic reviews suggesting superior quality over narrative reviews. In addition, the studies were conducted in inpatient, outpatient, emergency, etc., and multiple specialties were assessed, including medicine, surgery, pediatrics, gynecology, obstetrics, etc. The specialties were not limited to primary medical specialties and involved allied fields such as nursing, midwifery, and foundation courses. The participants’ education levels were also diverse, with the involvement of undergraduate students, postgraduate/specialty trainees, and even preceptors.

The oldest three articles on mini CEX were all authored by Norcini JJ and colleagues [1,13,22]. In the first article published, the authors found mini CEX to be satisfactory for evaluators and medicine trainees, with good reproducibility in a variety of settings [13]. The concerns raised were prolonged duration (>20 minutes), difficulty in administration for numerous trainees, and deviation from focused assessment. In their second study, the authors addressed the issue of examiners’ variations/stringency in mini-CEX assessment [22]. They observed no significant differences in examiners’ ratings in terms of examiners, training program, setting, and the nature of the patient. While the above two studies showed the effectiveness of mini-CEX in various settings, there have been concerns regarding the need for the large volume of students, evaluators, and settings. Therefore, the authors observed a large volume of internal medicine mini-CEX encounters [1]. The authors suggested that examiners overcompensate for patient difficulty considering that each resident interacts with several patients. Moreover, because multiple encounters are involved, there are chances of better performance and varying complexity with different encounters. This study was the top-cited research, with 411 citations [1]. The above three were landmark studies by Norcini JJ, after which several studies in different specialties suggested an acceptable nature of mini-CEX. The major addressed points included the number of encounters for reliable assessment, variation in assessment duration, objective and subjective evaluation of feedback, subjective variations in examiners’ evaluations, generalizability, validity, i.e., the ability to differentiate between different education levels, reliability (differentiate between poor vs. fair performance), and internal consistency [15-18,23]. These aspects were found satisfactory. Few studies investigated the mini-CEX-related perceptions of assessors and students/trainees [24-26]. While most participants appreciated the idea and benefits of mini-CEX, the concerns raised included limited interest among assessors, inadequate observation and feedback, time management, lack of standardization, prior mini-CEX-related training, and cost of implementation [24-26]. More studies on these aspects are needed to understand mini-CEX implementation better. An additional concern is that the mini-CEX implementation has mainly been investigated in the Internal Medicine specialty, and its role in several other specialties for undergraduates and postgraduates teaching is yet to be investigated.

While we attempted to perform a comprehensive bibliometric analysis of mini-CEX-related research, it is bound to have some limitations. First, the study counts the articles generated through one major database (Web of Science), and there might be minor variations compared to the results from other major databases. However, the database is widely recognized and should provide substantial inferences. Second, the citation count does not exclude self-citations, which could marginally affect the citation count. Third, the quantitative and qualitative information provided in the selected articles is vast, and it is beyond the scope of the current study to analyze every aspect of such information. Nevertheless, the specific quantitative information provided in the current analysis can help formulate future research and strengthen mini-CEX-related publications. Moreover, the results are mostly pictorial representations of the research trends that would make the understanding straightforward.

## Conclusions

The mini-CEX has been utilized as a teaching-assessment method in various specialties, clinical settings, and participants’ education levels. However, the volume of research conducted on mini-CEX is low. Additionally, the published articles have reduced impact and growth, as evidenced by low annual growth rate and citation trends, especially in recent years. However, the available evidence is of reasonable quality considering the contribution from mostly prospective studies. It suggests the huge potential for further investigating the role of mini-CEX in clinical teaching. More studies are needed to establish the positive impact of mini-CEX in medical education.
